# Synergy of polymyxin B and minocycline against KPC-3- and OXA-48-producing *Klebsiella pneumoniae* in dynamic time–kill experiments: agreement with *in silico* predictions

**DOI:** 10.1093/jac/dkad394

**Published:** 2023-12-30

**Authors:** Anna Olsson, Christer Malmberg, Chenyan Zhao, Lena E Friberg, Elisabet I Nielsen, Pernilla Lagerbäck, Thomas Tängdén

**Affiliations:** Department of Medical Sciences, Uppsala University, Uppsala, Sweden; Department of Medical Sciences, Uppsala University, Uppsala, Sweden; Department of Pharmacy, Uppsala University, Uppsala, Sweden; Department of Pharmacy, Uppsala University, Uppsala, Sweden; Department of Pharmacy, Uppsala University, Uppsala, Sweden; Department of Medical Sciences, Uppsala University, Uppsala, Sweden; Department of Medical Sciences, Uppsala University, Uppsala, Sweden

## Abstract

**Objectives:**

Combination therapy is often used for carbapenem-resistant Gram-negative bacteria. We previously demonstrated synergy of polymyxin B and minocycline against carbapenem-resistant *Klebsiella pneumoniae* in static time–kill experiments and developed an *in silico* pharmacokinetic/pharmacodynamic (PK/PD) model. The present study assessed the synergistic potential of this antibiotic combination in dynamic experiments.

**Methods:**

Two clinical *K. pneumoniae* isolates producing KPC-3 and OXA-48 (polymyxin B MICs 0.5 and 8 mg/L, and minocycline MICs 1 and 8 mg/L, respectively) were included. Activities of the single drugs and the combination were assessed in 72 h dynamic time–kill experiments mimicking patient pharmacokinetics. Population analysis was performed every 12 h using plates containing antibiotics at 4× and 8× MIC. WGS was applied to reveal resistance genes and mutations.

**Results:**

The combination showed synergistic and bactericidal effects against the KPC-3-producing strain from 12 h onwards. Subpopulations with decreased susceptibility to polymyxin B were frequently detected after single-drug exposures but not with the combination. Against the OXA-48-producing strain, synergy was observed between 4 and 8 h and was followed by regrowth. Subpopulations with decreased susceptibility to polymyxin B and minocycline were detected throughout experiments. For both strains, the observed antibacterial activities showed overall agreement with the *in silico* predictions.

**Conclusions:**

Polymyxin B and minocycline in combination showed synergistic effects, mainly against the KPC-3-producing *K. pneumoniae*. The agreement between the experimental results and *in silico* predictions supports the use of PK/PD models based on static time–kill data to predict the activity of antibiotic combinations at dynamic drug concentrations.

## Introduction

The global emergence and spread of carbapenem-resistant Gram-negative bacteria poses an urgent medical challenge.^1^ Treatment options are limited in severe infections caused by carbapenemase-producing isolates, due to their MDR phenotypes.^2,3^ Combination therapy is frequently prescribed to enhance the activity of the available antibiotics and improve patient outcomes.^4^ Polymyxin B and E (colistin) have been widely used and recommended as part of combination therapy including a second *in vitro* active antibiotic.^3,5^ In practice, polymyxin-based combinations remain last-resort treatments in cases where newer antibiotics (e.g. cefiderocol and carbapenemase inhibitor combinations) are unavailable or not appropriate due to resistance or intolerance.^2^

Polymyxins target the LPS structure in the outer membrane of Gram-negative bacteria and cause disruption of the bacterial outer membrane. A presumed mechanism of synergistic interactions with these antibiotics is that the disruption of the bacterial outer membrane increases the permeability of a second drug. By this mechanism, polymyxins may counteract resistance mediated by membrane changes, porin loss or active efflux.^5,6^ Minocycline is a semisynthetic tetracycline derivative, which is often active against MDR Gram-negative bacteria, in particular *Acinetobacter baumannii*, and has pharmacokinetic (PK) advantages compared with tigecycline.^7^ Minocycline interferes with protein synthesis through interaction with the 30S ribosomal subunit.^8^

We previously reported synergy with polymyxin B and minocycline against an OXA-48-producing *Klebsiella pneumoniae* strain, which was non-susceptible to both single antibiotics, in 24 h static time–kill experiments.^9^ Based on data obtained for this strain, we developed an *in silico* pharmacokinetic/pharmacodynamic (PK/PD) model.^10^ The model predicted the combination to be potentially clinically useful at drug exposures achieved in critically ill patients at conventional or higher dosing.

The objective of this study was to experimentally validate the synergistic potential of polymyxin B and minocycline against KPC- and OXA-48-producing *K. pneumoniae* at antibiotic concentration–time profiles mimicking patient PK. We performed 72 h dynamic time–kill experiments with repeated sampling for viable counts to assess the antibacterial activities of the single drugs and the combination. Emergence of resistance was assessed by population analysis performed every 12 h and WGS was used to characterize resistance genes and mutations. Finally, the observed antibacterial effects were compared with the predictions from the *in silico* PKPD model.

## Materials and methods

### Antibiotics and media

Antibiotics were purchased from Sigma–Aldrich (St. Louis, MO, USA). Stock solutions of 10 000 mg/L were prepared by dissolving polymyxin B and minocycline in sterile water. Cation-adjusted Mueller–Hinton (MH-II) (BD Diagnostics, Sparks, MD, USA) broth and agar were used in all experiments.

### Strains and antibiotic susceptibility testing

Two clinical carbapenemase-producing *K. pneumoniae* strains were used. The OXA-48-producing strain ARU613 was provided by the Public Health Agency of Sweden, and ARU705, producing KPC-3, was obtained from Erasmus MC in Rotterdam, the Netherlands. Antimicrobial susceptibilities were tested by broth microdilution according to CLSI recommendations and interpreted using CLSI clinical breakpoints.^11,12^

### Genetic characterization

The Epicentre MasterPure DNA purification Kit (Illumina Inc., San Diego, CA, USA) was used for DNA preparation prior to WGS using the MiSeq platform (Illumina Inc.), followed by *de novo* assembly by CLC Genomics Workbench version 21 (QIAGEN, Hilden, Germany). Acquired resistance genes were identified in contigs using the ResFinder database 4.0 and verified using CLC Main Workbench 21 (QIAGEN).^13^ A database including gene sequences of porins, efflux pumps and associated transcription regulators, as well as genes associated with polymyxin B resistance, was created using *K. pneumoniae* MGH 78578 (NCBI Reference Sequence NC_009648.1) as a reference strain. Alignments were created using CLC Main Workbench 21 (QIAGEN).

### PD predictions

The effects of single- and two-drug exposures of polymyxin B and minocycline in dynamic time–kill experiments were predicted using the previously developed *in silico* semi-mechanistic PK/PD model. This model was based on *in vitro* static time–kill curve data of ARU613 and quantified the individual and combined effects of polymyxin B and minocycline.^10^ Static time–kill experiments were performed with ARU705, as described below, to generate strain-specific drug-effect parameters for the model. The inoculum sizes in the predictions were set to 6 log_10_ cfu/mL. It was assumed that no bacteria could regrow if the total amount was predicted to be <1 cfu in the 100 mL working volume. Model fit and simulations were performed in NONMEM^®^ (version 7.4, Icon Development Solutions, Hanover, MD, USA) and mrgsolve (https://mrgsolve.github.io/).^14^

### Static time–kill experiments

Static time–kill experiments with ARU705 were performed as previously described.^10^ In short, bacterial starting inocula of 6 log_10_ cfu/mL were exposed to 11 2-fold increasing concentrations of the single antibiotics: polymyxin B at 0.0625–64 mg/L (0.125–128× MIC) and minocycline at 0.25–256 mg/L (0.25–256× MIC). The combined activity of the antibiotics was evaluated at nine different drug concentrations: polymyxin B was added to 0.25–1 mg/L and minocycline to 0.5–16 mg/L. The test tubes were incubated at 37°C with shaking at 190 rpm and samples for viable counts were collected at 0, 1, 2, 4, 8, 24 and 28 h. All experiments were performed in duplicate.

### In vitro PK/PD model

A previously described in-house *in vitro* PK/PD model was used for the dynamic time–kill experiments (Figure S1, available as Supplementary data at *JAC* Online).^15^ In this model, antibiotic-containing medium is drawn out of the bacterial compartment using a peristaltic pump (P-500, Pharmacia, Sweden). The diluent medium is simultaneously drawn into the bacterial compartment by negative pressure. A 0.45 μm membrane filter (#7000-0004, Cytiva, USA) protected by a glass-fibre prefilter (#F6036, Merck, USA) retains the bacteria in the compartment.

### Design of PK profiles

The PK profiles of polymyxin B and minocycline were designed to mimic the free concentration–time profiles of the antibiotics in the plasma of critically ill patients. For practical reasons, the PK profiles were designed based on injection of polymyxin B and minocycline rather than 1 h infusion, which is often used in clinical practice. The following dose regimens were mimicked, which correspond to standard dosing of polymyxin B and a high-dose regimen for minocycline: a loading dose (LD) of 2.5 mg/kg followed by a maintenance dose (MD) of 1.5 mg/kg every 12 h (q12h) for polymyxin B and an LD of 400 mg followed by an MD of 200 mg q12h for minocycline.^5,16^ Patient PK profiles were simulated based on two-compartment population PK models, applying a free fraction of 42% for polymyxin B and an equation for atypical non-linear concentration-dependent protein binding for minocycline.^10,17–19^

The targeted peak drug concentrations (*C*_max_) were 5.0 mg/L after the LD and 4.5 mg/L after q12h MDs for polymyxin B, and 3.8 mg/L after the LD and 4.1 mg/L after q12h MDs for minocycline (Figure S2). The targeted trough concentrations (*C*_min_) were 1.1 mg/L for polymyxin B and 1.8 mg/L for minocycline. The flow rates after each injection were set to obtain half-lives of 2 and 10 h for the first and second phases, respectively, to best match the two-compartment profiles of polymyxin B. The switch point between the two phases was 3 h after the first dose and 2 h after the following doses. Based on measured drug concentration in initial experiments with the single antibiotics, a compensation factor of ×1.67 was used for both drugs to compensate for surface binding and degradation. The diluent medium had a constant concentration of 1.2 mg/L minocycline to compensate for the longer half-life of minocycline: 3 and 22 h (on average) for the first and second phases, respectively. The area-under-the-concentration–time curves (AUCs) simulated for the *in vitro* experimental setup agreed (99%–102%) with those expected in a typical patient.

### Assessment of drug concentrations

To confirm that the desired PK profiles were achieved, samples were collected at multiple timepoints to determine the drug concentrations in duplicate experiments without bacteria. We applied a biological method using *Staphylococcus aureus* (ATCC 29213) as indicator organism for minocycline and a polymyxin B-susceptible clinical *Escherichia coli* isolate (ARU1051) for polymyxin B.^20^ The indicator organism was added to MH-II agar to an inoculum of 5 log_10_ cfu/mL and 80 mL agar aliquots were added into Petri dishes of 15 cm diameter. When solid, 5 mm wells were punched in the agar and 70 μL samples were added to each well. Zone diameters were measured after 15–18 h of incubation at 37°C. The method was validated by creating a standard curve, i.e. a linear function of measured zone diameters and ln(concentration) for polymyxin B (1, 2, 4 and 8 mg/L) or minocycline (0.25, 0.5, 1 and 2 mg/L), for each plate using R^2^ ≥ 0.90 as a cut-off. The mean R^2^ values were 0.981 (range 0.923–1.00) for polymyxin B and 0.991 (range 0.948–1.00) for minocycline.

### Dynamic time–kill experiments

Bacteria in exponential growth phase were added to achieve a starting inoculum of *c.* 6 log_10_ cfu/mL. Samples were collected at 0, 1, 2, 4, 6, 8, 12, 24, 25, 26, 28, 30, 32, 36, 48, 49, 50, 52, 54, 56, 60 and 72 h and plated on MH-II plates for viable counts. In addition, samples collected at 0 h and every 12 h throughout the experiments were plated on MH-II plates with polymyxin B or minocycline at 4× MIC and 8× MIC for population analysis profiling. Bacterial colonies were counted after 24 and 48 h of incubation. All experiments were performed in duplicate. One colony from each plate in the first replicate was subjected to MIC determination. When populations with an increased MIC to polymyxin B or minocycline were detected, a representative isolate was genetically characterized.

The following definitions were used in interpreting time–kill data: a bactericidal effect, ≥ 3 log_10_ reduction in cfu/mL compared with the starting inoculum; synergy, ≥ 2 log_10_ cfu/mL reduction with the combination compared with the most active single antibiotic; and antagonism, ≥1 log_10_ cfu/mL increase with the combination compared with the most active single antibiotic. Undetectable growth was counted as 1 log_10_ cfu/mL (lower limit of detection, LOD). If clogging of the filter occurred due to bacterial growth >8 log_10_ cfu/mL (upper limit of quantification, LOQ), the last measured value was used in the assessment of combination effects.

### Data availability

Raw data from sequencing are deposited in the NCBI Sequence Read Archive under BioProject accession numbers PRJNA947381 (ARU705) and PRJNA957334 (ARU613).

## Results

### Phenotypic and genetic characterization of bacterial strains

Both strains were resistant to meropenem (MICs ≥ 16 mg/L) and produced carbapenemases; ARU705 carried *bla*_KPC-3_ and ARU613 carried *bla*_OXA-48_ (Table 1). A frameshift in the gene encoding the porin OmpK35 was identified in both ARU705 (Q292fs) and ARU613 (L63fs), likely contributing to non-susceptibility (Table 2). Sequence variations were also identified in genes involved in AcrAB-TolC efflux, for which β-lactams and minocycline are known substrates.^21^ However, the biological impact of the identified sequence variations is unclear. The *tet*(D) gene was found in ARU613 (minocycline MIC 8 mg/L) but not in ARU705 (minocycline MIC 1 mg/L). This gene encodes an efflux pump conferring acquired resistance to tetracyclines, although usually not to minocycline.^22^

**Table 1.** MIC values, classification of antibiotic susceptibilities, identified resistance genes and amino acid variations

Antibiotic	Function	Gene	MIC (mg/L) and resistance genotype	
			ARU705	ARU613
Meropenem	Enzymatic inactivation		≥16 (R)	≥16 (R)
		*bla*_OXA-9_	+	+
		*bla*_OXA-48_	−	+
		*bla*_CTX-M-14b_	−	+
		*bla*_KPC-3_	+	−
		*bla*_SHV-28_	+	+
		*bla*_SHV-106_	+	+
		*bla*_TEM-1A_	+	−
		*bla*_TEM-1C_	−	+
Minocycline	Efflux		1 (S)	8 (I)
		*tet*(D)	−	+
			0.5 (I)	8 (R)
Polymyxin B	Drug target modifications	*phoP*	+	+
		*phoQ*	+	+
		*mgrB*	+	C39S
		*pmrA*	+	A217V
		*pmrB*	+	T246A
		*crrA*	−	−
		*crrB*	−	−
		*lpxM*	S253G	S253G

S, susceptible; I, intermediate; R, resistant; +, full-length gene present; −, gene not found.

**Table 2.** Sequence variations in genes encoding efflux pumps and porins

Efflux pump or porin	Function	Gene	ARU705	ARU613
AcrAB-TolC	S	*acrA*	+	+
	S	*acrB*	+	+
	R	*acrZ*	+	+^a^
	R	*acrR*	+	+
	S	*tolC*	N73T I82V E203G N251S S271N S276T I278V S282R S284N H289Del N291T Q293L Q294A Q296N N298S A300N	N73T I82V E203G N251S S271N S276T I278V S282R S284N H289Del N291T Q293L Q294A Q296N N298S A300N
	A	*marR*	+	+
	A	*marA*	+	+
	A	*marB*	+	+
	A	*rob*	+	+
	R	*ramR*	G151D	+^a^
	A	*ramA*	−	+^a^
	R	*soxR*	+	+
	R	*soxS*	+	L60R
OmpK35	S	*ompK35*	Q292fs	L63fs
OmpK36	S	*ompK36*	P178V L184Del S185Del P186Del T192G A193W L194S Y201F Y210W N221H L225T G226D D227E K231V L232P T258S	Ins136G Ins137D P180V L186Del S187Del P188Del T194G A195W L196S Y203F Y212W N223H L227T G228D D229E K233V L234P T260S Ins311R
OmpA	S	*ompA*	+	+
OqxAB	S	*oqxA*	+	T341I
	S	*oqxB*	K148N G540S Y783F P1049L	K148N G540S
	A	*rarA*	Q99fs	+
	R	*oqxR*	R69fs	+

S, subunit; A, activator; R, repressor; Del, deletion; fs, frameshift; Ins, insertion. *K. pneumoniae* MGH 78578 (NCBI accession number: NC_009648) was used as reference; ‘+’ indicates 100% similarity to the reference gene sequence.

^a^Identification confirmed using read mapping.

Polymyxin resistance is commonly caused by LPS modifications.^6^ The PmrAB and CrrAB two-component systems regulate L-Ara4N modifications of lipid A, which reduce the negative charge of the polymyxin B target. In ARU613, polymyxin B resistance (MIC 8 mg/L) was probably due to a mutation in *pmrB* (T246A), which has previously been reported.^23,24^ However, an *mgrB* mutation (C39S) was also identified. A mutation in *pmrA* (A217V) was detected in ARU613 but was not predicted to cause resistance.^25^ Both strains also carried a mutation in *lpxM* (S253G), which was associated with colistin resistance in a previous study; yet, the impact of the *lpxM* mutation alone is unclear.^26^

### Static time–kill experiments and PK/PD model parameters for ARU705

**Figure 1.**
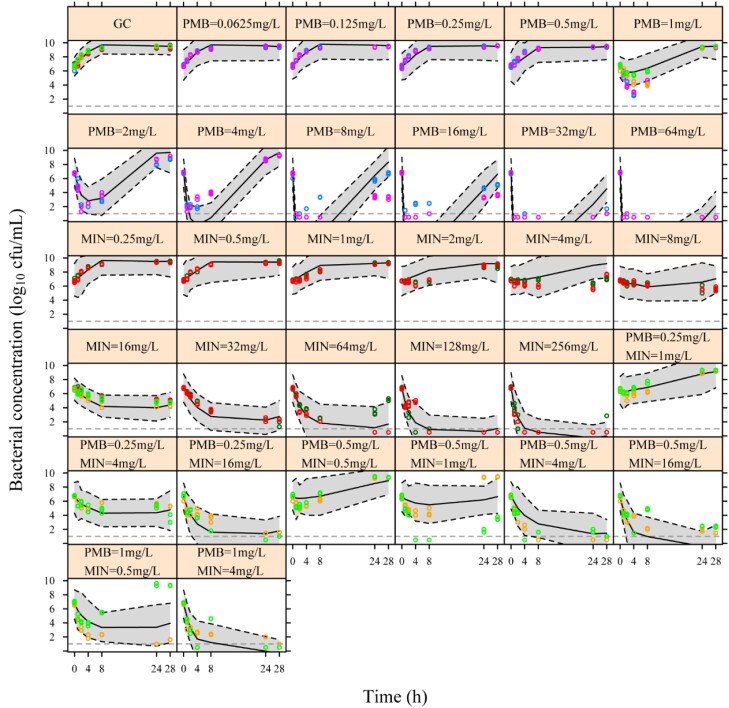
Static time–kill data for ARU705 and visual predictive checks for the PK/PD model. The measured bacterial concentrations are shown as open circles (colours indicate individual experiments). The median (solid lines) and 95% CI of the median (grey shade and black dashed lines) are defined from the datasets simulated from the model. Horizontal dashed lines indicate the LOD of 1 log_10_ cfu/mL, and observations below LOD (no detectable growth) are presented as 0.5 log_10_ cfu/mL. GC, growth control; MIN, minocycline; PMB, polymyxin B.

Static time–kill experiments with ARU705 showed synergy with the combination at antibiotic concentrations of polymyxin B ranging from 0.25 to 1 mg/L and minocycline at 0.5–16 mg/L (Figure 1). Rapid initial killing followed by regrowth was observed with polymyxin B at concentrations of 1–16 mg/L. The results were used to generate PK/PD model parameters to inform the *in silico* PD predictions of this strain (Table 3 and Table S1).

**Table 3.** PK/PD model parameters used for *in silico* PD predictions^a^

Parameters	Description	Parameter values
Bacteria-related		
*k_growth_* (h^−1^)	Rate constant of bacterial growth	1.37
*k_death_* (h^−1^)	Rate constant of natural bacterial death	0.179
*B_max_* (cfu/mL)	Maximum bacterial concentration in system	10^9.53^
*T_lag_* (h)	Lag time for bacteria to transfer from S to R	0.304

		MIN		PMB	
Single drug-specific		ARU613	ARU705^b^	ARU613	ARU705^b^
*Slope_1_* (L/mg/h)	Slope for power function of *k_drug_*	0.339	1.55 (6.2%)	0.0690	57.1 (3.9%)
*Slope_2_* (L/mg/h)	Slope for linear function of *k_on_*	0.000179	2.23 (15%)	0.00402	50.4 (3.9%)
*ɤ_1_* (−)	Exponent for power function of *k_drug_*	0.546		1.20	
*Slope_3_* (−)	Slope for linear function for AR	−10.2		1	

Combination-specific interaction (PMB affecting MIN)		ARU613	ARU705^b^
*E_max_* (−)	*E*_max_ for interaction function of *k_drug,MIN,COMB_*	2.45	—
*EC_50_* (mg/L)	EC_50_ for interaction function of *k_drug,MIN,COMB_*	0.285	0.553 (13%)
*Slope_4_* (−)	Slope for interaction function of *k_on,MIN,COMB_*	8.32	—
*ɤ_4_* (−)	Exponent for interaction function of *k_on,MIN,COMB_*	0.479	—

S, susceptible compartment; R, resting compartment; MIN, minocycline; PMB, polymyxin B; AR, adaptive resistance; COMB, combination.

^a^Model structures can be found in the report by Zhao *et al.*^10^

^b^Re-estimated parameters for ARU705 are expressed as a scaling factor of the corresponding values for ARU613 and relative standard error (in parentheses). For example, the estimated Slope_1_ for ARU705 for MIN single drug is 0.339 × 1.55 = 0.525.

### Dynamic time–kill experiments

**Figure 2.**
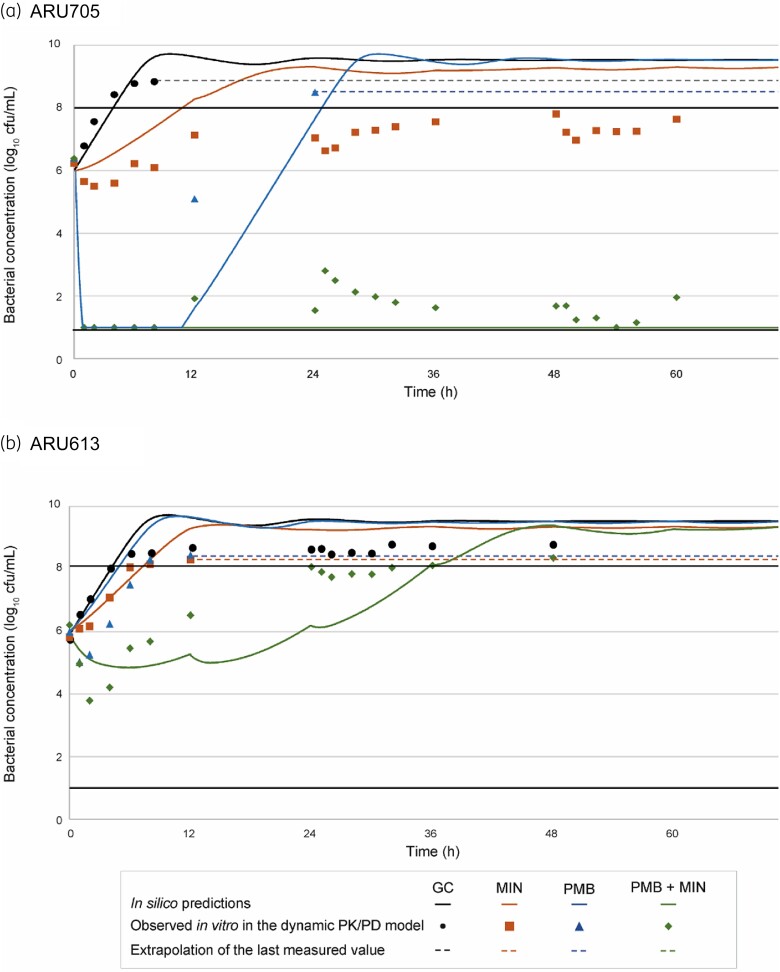
Predicted and measured mean bacterial concentrations in the dynamic time–kill experiments. The black lines depict the LOD (1 log_10_ cfu/mL) and the LOQ (8 log_10_ cfu/mL). When clogging of the filter occurred due to high bacterial concentrations, the last measured value is extrapolated with a dashed line. GC, growth control; MIN, minocycline; PMB, polymyxin B.

Based on visual inspection, we considered the agreement between the targeted and measured drug concentrations in the dynamic time–kill experiments satisfactory (Figure S2). Overall, the *in silico* predictions adequately captured the observed antibacterial activities of the single antibiotics and the combination (Figure 2). Polymyxin B and minocycline in combination showed synergistic and bactericidal effects against ARU705 starting at 12 h and throughout the 72 h experiments (Figure 2a and Table S2). Against this strain, polymyxin B alone resulted in rapid initial killing, followed by regrowth after 8 h. Minocycline alone resulted in limited initial killing and was also followed by regrowth; however, bacterial concentrations were reduced compared with the growth control and remained between approximately 5.5 and 8 log_10_ cfu/mL over 72 h. The combination was less effective against ARU613, which was non-susceptible to minocycline and polymyxin B. Against this strain, no significant bacterial killing was observed with single-drug exposures and synergy with the combination was observed only at 4 to 8 h (Figure 2b and Table S3).

### Population analysis profiling

**Figure 3.**
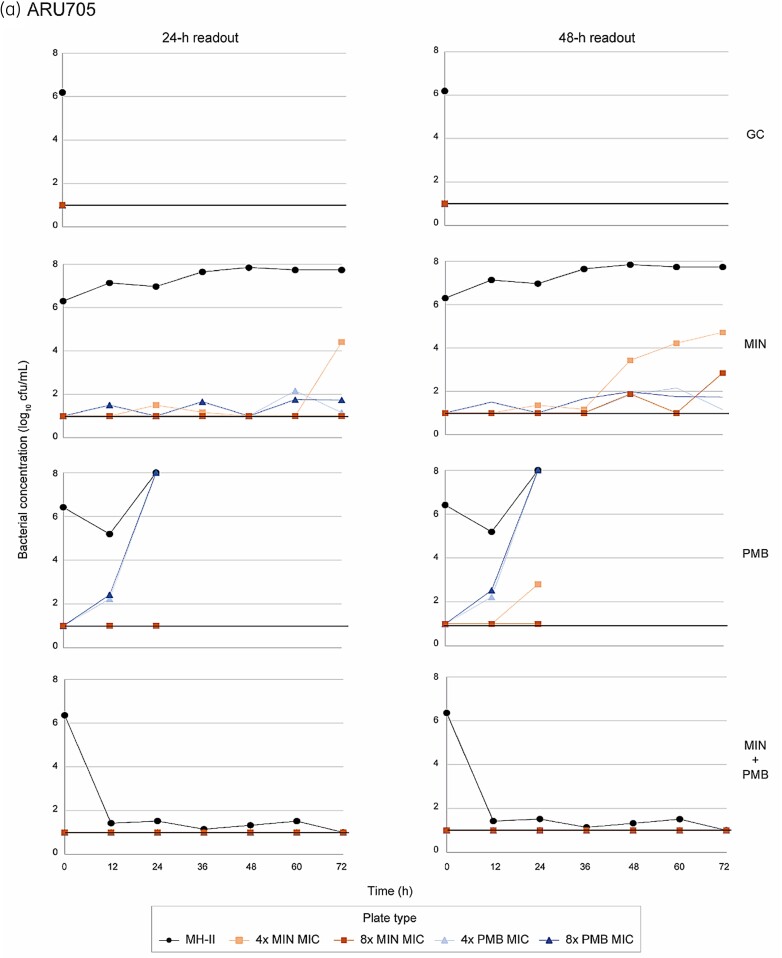

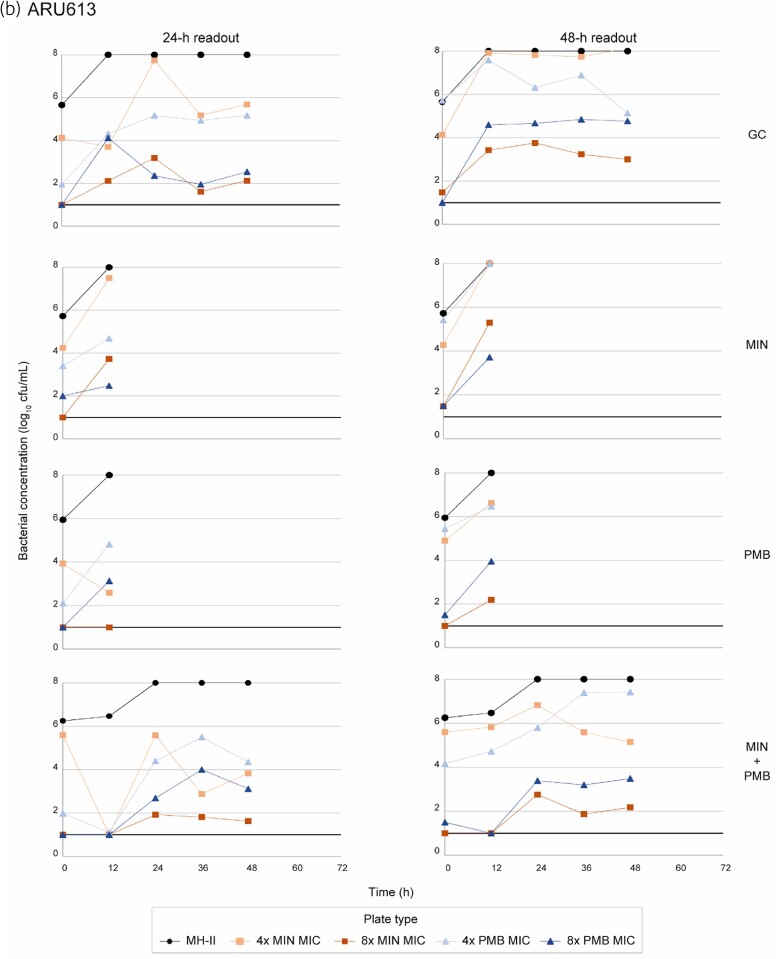
Population analysis profiling during dynamic time–kill experiments. Viable counts (mean log_10_ cfu/mL) on regular plates and plates containing minocycline or polymyxin B at 4 × or 8 × MIC with 24 h and 48 h readout are presented. GC, growth control; MIN, minocycline; PMB, polymyxin B.

In experiments with the single antibiotics against ARU705, we detected subpopulations with reduced susceptibility to polymyxin B after 12 h and subpopulations with reduced susceptibility to minocycline after 24 h (Figure 3a). Within 24 h of exposure to polymyxin B alone, the bacterial growth on polymyxin B plates was equal to the growth on plates that did not contain antibiotics. As expected, more colonies were detected when plate incubation was extended from 24 to 48 h (up to 4 log_10_ cfu/mL increase). Six isolates growing on polymyxin B plates at 1.3 to >8 log_10_ cfu/mL were subjected to MIC determination, which showed 32- to 64-fold increases in polymyxin B MIC. Only 2- to 8-fold increases in MIC were found in six isolates growing on minocycline plates at 2.34–3.26 log_10_ cfu/mL. No growth on antibiotic-containing plates was detected after exposure to the combination.

With ARU613, emergence of resistance was more frequent and subpopulations with reduced susceptibility were present already at 0 h (Figure 3b). With this strain, ≥8-fold increases in polymyxin B MICs were found in 38/43 (88%) of the tested isolates growing on polymyxin B plates. At least 8-fold higher minocycline MICs were observed in 18/38 (47%) tested isolates growing on minocycline. Also, extending the incubation time from 24 to 48 h revealed additional bacterial growth for ARU613 (up to 6 log_10_ cfu/mL increase).

### Genetic characterization of emerging resistant isolates

After the experiments, WGS of isolates with increased MIC revealed several genetic alterations compared with the parental strains. Sequencing revealed several mutations in genes affecting polymyxin B susceptibility and that up to three subpopulations were often present in the same sample (Tables S4 and S5). For ARU705, a frameshift in *mgrB* (K3fs) was identified in isolates growing at 8 × and 4 × polymyxin B MIC after 36 and 48 h of exposure to minocycline alone. Mutations were also identified after polymyxin B monotherapy in *pmrB* (T157P) and in *phoP* (L12Q) in separate isolates from the same 24 h sample. We identified a frameshift in *mgrB* in an isolate with a 64-fold increase in polymyxin B MIC, and mutations in *phoP* (L12Q) or *pmrB* (T157P) in three isolates with 32–64-fold polymyxin B MIC elevations (MICs 16–32 mg/L). The sequence variation *lpxM* (S253G) was shared by the parental strain and the resistant isolates and was not found in the reference strain.

For ARU613, emerging mutations were found in *phoP* (I63F) and *phoQ* (A22T and P305T) in four isolates growing on polymyxin B plates after minocycline or polymyxin B monotherapy, all of which had 8–32-fold increases in polymyxin B MIC (64–256 mg/L). The latter mutations are not previously known to confer resistance to polymyxin B. A *phoP* mutation (S128P) was found in 1 of 8 sequenced resistant isolates detected after exposure to the two-drug combination. Sequence variations in the parental strain and resistant isolates, compared with the reference strain, were detected in *pmrA* (A217V), *pmrB* (T246A), *mgrB* (C39S) and *lpxM* (S253G).

## Discussion

In this study, we validated the synergistic activity of polymyxin B and minocycline against KPC- and OXA-48-producing *K. pneumoniae* at antibiotic concentration–time profiles mimicking patient PK. The experimental design was informed by a previously developed PK/PD model based on static time–kill data with the OXA-48-producing *K. pneumoniae* ARU613, which predicted that polymyxin B and minocycline in combination would show synergistic but not bactericidal effects at drug concentrations achieved in critically ill patients.^10^ We added a KPC-3-producing strain (ARU705), which had lower MICs of the tested antibiotics, and performed static time–kill experiments to generate strain-specific drug-effect parameters. Both strains were then exposed to polymyxin B and minocycline, alone and in combination, at drug concentrations mimicking patient PK for polymyxin B at standard dosing (2.5 mg/kg LD followed by 1.5 mg/kg q12h) and a high-dose regimen of minocycline (400 mg LD followed by 200 mg q12h).

Against ARU705, the combination demonstrated synergy and a bactericidal effect at 12 h, which was sustained during the 72 h dynamic time–kill experiments. This strain was classified susceptible (S) to minocycline and intermediate (I) to polymyxin B. As expected, the combination was less successful in the experiments with ARU613, which was non-susceptible to both antibiotics (MICs 8 mg/L); synergy was observed at 4–8 h but was followed by regrowth. In the previous static time–kill study, synergy after 24 h was observed with polymyxin B at 2 mg/L in combination with minocycline at 4 mg/L.^10^ In the dynamic experiments mimicking patient PK, the polymyxin B concentration was <2 mg/L after 4 h and the minocycline concentration was <4 mg/L already 1 h after injection.

We recently reported synergistic and bactericidal effects of polymyxin B and minocycline at static concentrations against 18/20 carbapenemase-producing *K. pneumoniae* in a larger screening.^27^ All strains were susceptible to minocycline, but nine strains were resistant to polymyxin B, which did not preclude synergistic effects. Moreover, positive interactions have been demonstrated against carbapenemase-producing *E. coli*, also in the presence of *tet*(B), which encodes a minocycline efflux pump, and against MDR *Pseudomonas aeruginosa* and *A. baumannii*.^28–30^ With this combination, synergy can be achieved by polymyxin-induced damage to the bacterial outer membrane, resulting in increased permeability and decreased efflux of minocycline.

The two original strains harboured several genes and mutations that may confer carbapenem resistance by β-lactamase production, porin alteration and active efflux. No specific genes or mutations previously known to cause minocycline resistance were detected; however, sequence variations were found in genes involved in AcrAB-TolC efflux. We also identified a mutation in *pmrB* (T246A) in ARU613, which may result in L-Ara4N modifications of lipid A regulated by PmrAB.^6,23^ Although the effects on resistance levels by this mutation are not fully understood, it is likely to counteract target binding and membrane disruption, thereby decreasing the activity and synergistic potential of polymyxin B.^23,24^

Population analysis profiling revealed subpopulations of ARU705 with reduced susceptibility within 12–24 h of exposure to either of the single antibiotics. Yet, no resistance development was detected during exposure to the combination. Emergence of resistance was more frequent in ARU613. With this strain, subpopulations with reduced susceptibility were revealed already before start of experiments, which indicates that there were pre-existing resistant subpopulations. Eight-fold or higher increases in MIC values were commonly detected in isolates growing on polymyxin B plates. In contrast, approximately half of the isolates growing on minocycline plates showed no increase in MIC values compared with the original strains, which suggests an inoculum effect, adaptive resistance or tolerance/persistence rather than selection of resistant subpopulations. For both strains, extending the incubation time from 24 to 48 h resulted in higher bacterial counts, probably due to slow growth of subpopulations affected by antibiotic exposure or mutations resulting in reduced fitness.

There was overall agreement between the results of the dynamic time–kill experiments compared with the *in silico* predictions. Most importantly, the PK/PD model accurately predicted synergy against both strains and a bactericidal effect against ARU705 with the two-drug combination, while bacterial killing was limited with both single antibiotics. This observation aligns with other studies reporting that the activity of dynamic antibiotic exposures can be adequately predicted based on static time–kill data.^31,32^ However, some discrepancies existed, especially in bacterial counts close to the limits of detection and quantification.

The only adjustment made when translating the generated 24 h static time–kill data to *in silico* predictions of dynamic time–kill experiments was to let concentrations predicted from patient PK models, rather than static antibiotic concentrations, determine the expected strain-dependent effect. Challenges to this approach include, for example, differences in growth conditions between static and dynamic time–kill experiments. As higher working volumes are used in dynamic compared with static experiments (100 versus 4 mL), pre-existing resistant subpopulations or emerging resistant mutants are more likely to appear. Also, late regrowth or emergence of resistance occurring in 72 h dynamic time–kill experiments may be difficult or impossible to predict based on 24 h static time–kill data. Nevertheless, our results are encouraging and indicate the usefulness of *in silico* PK/PD models to quantitatively describe antibiotic effects in experiments with different setups. When applying *in silico* predictions to design dynamic time–kill experiments, the workload is reduced as only selected experiments to verify model predictions are required. In turn, adding the generated data on bacterial growth and time-dependent emergence of resistance at dynamic drug concentrations informs predictions of which treatment regimens are suitable for *in vivo* and clinical evaluation.

This study adds to the previous data from static time–kill experiments in that dynamic antibiotic concentrations mimicking patient PK were used and the duration of experiments was extended from 24 to 72 h. The population analysis profiling and extensive genetic characterization of the original strains and emerging resistant isolates using WGS are strengths of the study. We acknowledge that the activity of single antibiotics and combinations are strain dependent and, consequently, the synergistic and bactericidal effects observed in this study may not apply to other carbapenemase-producing *K. pneumoniae* isolates with similar MIC values. As for all *in vitro* models, translation to the clinical situation is hampered for several reasons, such as differences in growth conditions and the lack of immune system effects.

In conclusion, polymyxin B and minocycline in combination showed synergistic potential against OXA-48- and KPC-3-producing *K. pneumoniae*. A sustained bactericidal effect and suppression of resistance were found against the KPC-3-producing strain. This combination should be further explored and might be useful as a last-resort treatment for MDR Gram-negative bacteria. Our results also support the use of *in silico* PK/PD models based on static time–kill data to predict the antibacterial activity of dynamic antibiotic concentrations. This approach can inform efficient study designs for the resource-demanding dynamic time–kill experiments as well as *in vivo* or clinical studies.

## Supplementary Material

dkad394_Supplementary_Data

## Supplementary data

Figures S1 and S2 and Tables S1 to S5 are available as Supplementary data at *JAC* Online.
