# Supplementary material for: Synergy of polymyxin B and minocycline against KPC-3- and OXA-48-producing Klebsiella pneumoniae in dynamic time–kill experiments: agreement with in silico predictions
Source: J Antimicrob Chemother. 2023 Dec 30;79(2):391–402. doi: 10.1093/jac/dkad394 (PMC10832586; doi:10.1093/jac/dkad394)
Supplement: dkad394_Supplementary_Data [file dkad394_supplementary_data.pdf]

Table S1. Equations defining the *in silico* pharmacokinetic-pharmacodynamic (PKPD) model. Parameter definitions are provided in Table 3.

Eq. 1	$\frac{dS}{dt} = k_{growth} \cdot S - \left(k_{death} + k_{drug,MIN} \cdot Inh_{AR,MIN} + k_{drug,PMB} \cdot Inh_{AR,PMB}\right) \cdot S - k_{SR} \cdot S$
Eq. 2	$\frac{dR}{dt} = k_{SR} \cdot S - k_{death} \cdot R$
Eq. 3	$k_{SR} = \frac{S+R}{B_{max}} \cdot (k_{growth} - k_{death})$, k_{SR}=0 if Time < Tlag
Eq. 4	$k_{drug} = Slope_1 \cdot C^{\gamma 1}$
Eq. 5	$k_{drug,MIN,COMB} = Slope_1 \cdot {C_{MIN}}^{\gamma 1} \cdot (1 + \frac{E_{max} \cdot C_{PMB}}{EC_{50} + C_{PMB}})$
Eq. 6	$Inh_{AR} = (1 - Slope_3 \cdot AR_{ON})^{\beta}$, β set to 1 for PMB or -1 for MIN
Eq. 7	$\frac{dAR_{ON}}{dt} = k_{on} \cdot AR_{OFF}$
Eq. 8	$\frac{dAR_{OFF}}{dt} = -k_{on} \cdot AR_{OFF}$
Eq. 9	$k_{on} = Slope_2 \cdot C$
Eq. 10	$k_{on,MIN,COMB} = Slope_2 \cdot C_{MIN} \cdot (1 + \ Slope_4 \cdot {C_{PMB}}^{r4})$

Abbreviations: Eq., equation; PMB polymyxin B; MIN, minocycline.

Table S2. Dynamic time-kill experiments with *Klebsiella pneumoniae* ARU705. Mean bacterial concentrations (log_{10} cfu/mL) and standard deviations (in parenthesis) are presented. Bacterial counts below the lower limit of detection are presented as 1 log_{10} cfu/mL and "-" indicates that no data is available. The difference in growth with the combination compared to the most effective single antibiotic (Δ) is depicted. A bactericidal effect is marked in bold and synergy is highlighted in grey. Underlined SD values highlights timepoints with a single replicate.

Time (h)	GC	MIN	PMB	PMB + MIN	△
0	6.29 (0.12)	6.23 (0.01)	6.40 (0.05)	6.39 (0.01)	0.16
1	6.79 (0.01)	5.66 (0.10)	**1.00** (0.00)	**1.00** (0.00)	0.00
2	7.57 (0.04)	5.51 (0.03)	**1.00** (0.00)	**1.00** (0.00)	0.00
4	8.43 (0.08)	5.61 (0.09)	**1.00** (0.00)	**1.00** (0.00)	0.00
6	8.79 (0.07)	6.23 (0.11)	**1.00** (0.00)	**1.00** (0.00)	0.00
8	8.34 (0.00)	6.10 (0.30)	**1.00** (0.00)	**1.00** (0.00)	0.00
12	-	7.14 (0.42)	5.10 (0.36)	**1.92** (0.92)	-3.19
24	-	7.05 (0.80)	8.51 (0.01)	**1.54** (0.54)	-5.51
25	-	6.64 (0.46)	-	**2.81** (1.81)	-3.84
26	-	6.73 (0.37)	-	**2.50** (1.50)	-4.24
28	-	7.24 (0.09)	-	**2.13** (1.13)	-5.11
30	-	7.29 (0.07)	-	**1.98** (0.98)	-5.31
32	-	7.40 (0.20)	-	**1.80** (0.80)	-5.61
36	-	7.57 (0.05)	-	**1.63** (0.63)	-5.94
48	-	7.81 (0.10)	-	**1.69** (0.69)	-6.13
49	-	7.24 (0.06)	-	**1.70** (0.09)	-5.55
50	-	6.97 (0.02)	-	**1.24** (0.24)	-5.73
52	-	7.29 (0.07)	-	**1.31** (0.31)	-5.99
54	-	7.25 (0.04)	-	**1.00** (0.00)	-6.25
56	-	7.26 (0.06)	-	**1.16** (0.16)	-6.11
60	-	7.65 (0.05)	-	**1.96** (0.96)	-5.69
72	-	7.78 (0.03)	-	**1.00** (0.00)	-6.78

Abbreviations: GC, growth control; MIN, minocycline; PMB, polymyxin B.

Table S3. Dynamic time-kill experiments with *Klebsiella pneumoniae* ARU613. Mean bacterial concentrations (log_{10} cfu/mL) and standard deviations (in parenthesis) are presented. Bacterial counts below the lower limit of detection are presented as 1 log_{10} cfu/mL and "-" indicates that no data is available. The difference in growth with the combination compared to the most effective single antibiotic (∆) is depicted. A bactericidal effect is marked in bold, and synergy is highlighted in grey. Underlined SD values highlights timepoints with a single replicate.

Time (h)	GC	MIN	PMB	PMB + MIN	△
0	5.74 (0.05)	5.82 (0.02)	5.99 (0.15)	6.21 (0.03)	0.39
1	6.54 (0.02)	6.10 (0.29)	5.04 (0.26)	4.98 (0.01)	-0.06
2	7.04 (0.01)	6.17 (0.66)	5.27 (0.14)	3.79 (0.05)	-1.48
4	8.01 (0.08)	7.09 (0.61)	6.25 (0.17)	4.22 (0.97)	-2.03
6	8.49 (0.01)	8.06 (0.02)	7.51 (0.16)	5.47 (1.29)	-2.04
8	8.51 (0.03)	8.16 (0.11)	8.31 (0.09)	5.69 (1.15)	-2.47
12	8.68 (0.14)	8.30 (<u>0.00</u>)	8.44 (0.04)	6.53 (0.65)	-1.78
24	8.63 (0.07)	-	-	8.08 (0.04)	8.08
25	8.65 (0.11)	-	-	7.91 (0.44)	7.91
26	8.47 (0.08)	-	-	7.75 (0.17)	7.75
28	8.52 (0.00)	-	-	7.84 (0.12)	7.84
30	8.49 (<u>0.00</u>)	-	-	7.84 (0.06)	7.84
32	8.78 (<u>0.00</u>)	-	-	8.05 (0.22)	8.05
36	8.72 (<u>0.00</u>)	-	-	8.12 (0.10)	8.12
48	8.77 (<u>0.00</u>)	-	-	8.36 (0.15)	8.36
49	-	-	-	-	-
50	-	-	-	-	-
52	-	-	-	-	-
54	-	-	-	-	-
56	-	-	-	-	-
60	-	-	-	-	-
72	-	-	-	-	-

Abbreviations: GC, growth control; MIN, minocycline; PMB, polymyxin B.

Table S4. Characteristics of selected isolates growing on antibiotic plates in the population analysis of ARU705 after exposure to minocycline or polymyxin B in the dynamic time-kill experiments. Bacterial growth (log_{10} cfu/mL) on antibiotic-containing plates is indicated, as well as fold increases in MIC values and detected polymyxin B resistance genes and mutations. *Klebsiella pneumoniae* MGH 78578 (NCBI accession number: NC_009648) was used as reference and "+" indicates 100% similarity to the reference gene sequence.

Isolate	Antibiotic regimen	Time-point (h)	Plate	Bacterial growth	PMB MIC increase	MIN MIC increase	Resistance genes and mutations							
							phoP	*phoQ*	*pmrA*	*pmrB*	*mgrB*	*lpxM*	*crrA*	*crrB*
ARU705	-	-	-	-	-	-	+	+	+	+	+	S253G	-	-
1	MIN	36	8x PMB MIC	1.30	x64	x1	+	+	+	+	K3fs	S253G	-	-
2	MIN	48	4x PMB MIC	1.65	x64	x1	+	+	+	+	K3fs	S253G	-	-
3	PMB	24	4x PMB MIC	>8	x32	x1	+	+	+	T157P	+	S253G	-	-
4	PMB	24	8x PMB MIC	>8	x64	x2	L12Q	+	+	+	+	S253G	-	-
5	PMB	24	4x MIN MIC	2.30	x64	x8	L12Q	+	+	+	+	S253G	-	-

Abbreviations: GC, growth control; MIN, minocycline; PMB, polymyxin B; fs, frameshift.

Table S5. Characteristics of selected isolates growing on antibiotic plates in the population analysis of ARU613 after exposure to minocycline or polymyxin B in the dynamic time-kill experiments. Bacterial growth (log_{10} cfu/mL) on antibiotic-containing plates is indicated, as well as fold increases in MIC values and detected polymyxin B resistance genes and mutations. *Klebsiella pneumoniae* MGH 78578 (NCBI accession number: NC_009648) was used as reference and "+" indicates 100% similarity to the reference gene sequence.

Isolate	Antibiotic regimen	Time-point (h)	Plate	Bacterial growth	PMB MIC increase	MIN MIC increase	Resistance genes and mutations							
							phoP	*phoQ*	*pmrA*	*pmrB*	*mgrB*	*lpxM*	*crrA*	*crrB*
ARU613	-	-	-	-	-	-	+	+	A217V	T246A	C39S	S253G	-	-
144A	GC	48	4x PMB MIC	5.14	x8	x1	+	+	A217V	T246A	C39S	S253G	-	-
145A	GC	48	8x PMB MIC	4.76	x16	x2	+	+	A217V	T246A	C39S	S253G	-	-
145B	GC	48	8x PMB MIC	4.76	x16	x2	+	+	A217V	T246A	C39S	S253G	-	-
146A	GC	48	4x MIN MIC	8.13	x4	x8	+	+	A217V	T246A	C39S	S253G	-	-
147A	GC	48	8x MIN MIC	3.00	x2	x8	+	+	A217V	T246A	C39S	S253G	-	-
75	MIN	12	4x PMB MIC	7.97	x8	x2	+	A22T	A217V	T246A	C39S	S253G	-	-
76A	MIN	12	8x PMB MIC	3.71	x16	x2	I63F	+	A217V	T246A	C39S	S253G	-	-
76B	MIN	12	8x PMB MIC	3.71	x16	x4	+	+	A217V	T246A	C39S	S253G	-	-
80	PMB	12	4x PMB MIC	7.48	x8	x2	+	+	A217V	T246A	C39S	S253G	-	-
81A	PMB	12	8x PMB MIC	3.83	x32	x2	I63F	+	A217V	T246A	C39S	S253G	-	-
81B	PMB	12	8x PMB MIC	3.83	x32	x2	+	P305T	A217V	T246A	C39S	S253G	-	-
82	PMB	12	4x MIN MIC	6.80	x8	x8	+	+	A217V	T246A	C39S	S253G	-	-
83	PMB	12	8x MIN MIC	2.24	x4	x16	+	+	A217V	T246A	C39S	S253G	-	-
149A	PMB + MIN	48	4x PMB MIC	6.90	x16	x2	+	+	A217V	T246A	C39S	S253G	-	-
149B	PMB + MIN	48	4x PMB MIC	6.90	x16	x2	S128P	+	A217V	T246A	C39S	S253G	-	-
150A	PMB + MIN	48	8x PMB MIC	3.57	x32	x2	+	+	A217V	T246A	C39S	S253G	-	-
150B	PMB + MIN	48	8x PMB MIC	3.57	x32	x2	+	+	A217V	T246A	C39S	S253G	-	-
151A	PMB + MIN	48	4x MIN MIC	3.93	x2	x16	+	+	A217V	T246A	C39S	S253G	-	-
151B	PMB + MIN	48	4x MIN MIC	3.93	x4	x8	+	+	A217V	T246A	C39S	S253G	-	-
152A	PMB + MIN	48	8x MIN MIC	2.25	x4	x16	+	+	A217V	T246A	C39S	S253G	-	-
152B	PMB + MIN	48	8x MIN MIC	2.25	x8	x16	+	+	A217V	T246A	C39S	S253G	-	-

Abbreviations: GC, growth control; MIN, minocycline; PMB, polymyxin B; fs, frameshift.

A.

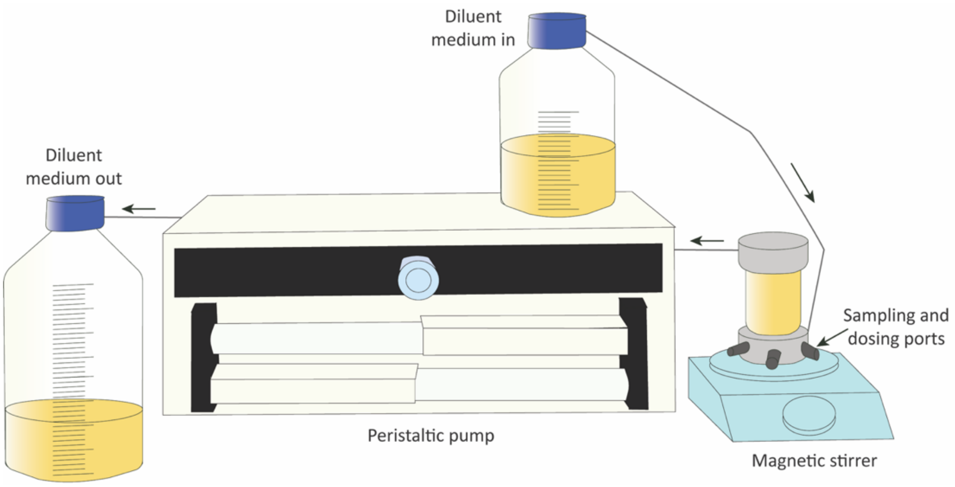

B.

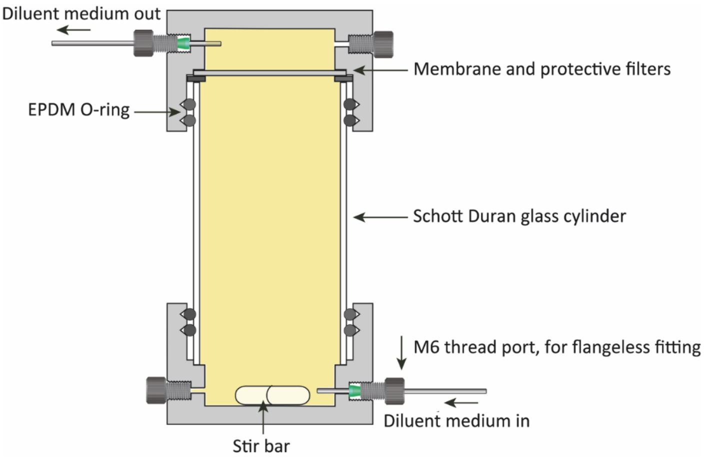

Abbreviations: EPDM, ethylene propylene diene monomer rubber.

Figure S1. Schematic illustration of the *in vitro* model used for dynamic time-kill experiments (A) and details of the bacterial compartment (B).

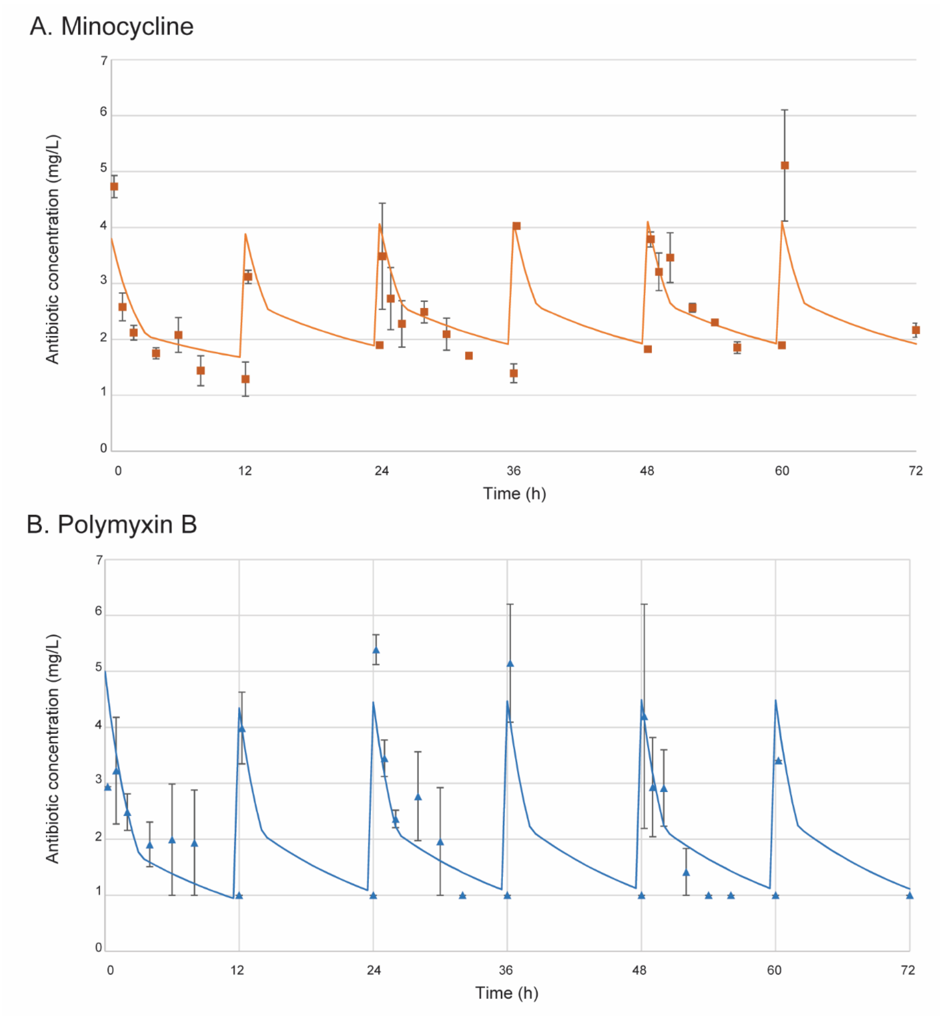

Figure S2. Targeted (lines) and measured (squares/triangles indicate mean concentrations and error bars indicate standard deviations) concentrations of minocycline and polymyxin B in the dynamic time-kill experiments.
